# The Impact of Vascular Anatomic Variations in the Infra-Pyloric Area on the Surgical Outcomes of Laparoscopic Pylorus-Preserving Gastrectomy in Early Gastric Cancer: A Post Hoc Analysis of a Multicenter Prospective Trial (KLASS-04)

**DOI:** 10.3390/jcm14072508

**Published:** 2025-04-07

**Authors:** Sang Soo Eom, Sin Hye Park, Young Shick Rhee, Sa-Hong Kim, Hyuk-Joon Lee, Young-Woo Kim, Han-Kwang Yang, Do Joong Park, Sang Uk Han, Hyung-Ho Kim, Woo Jin Hyung, Ji-Ho Park, Yun-Suhk Suh, Oh-Kyung Kwon, Wook Kim, Young-Kyu Park, Hong Man Yoon, Sang-Hoon Ahn, Seong-Ho Kong, Keun Won Ryu

**Affiliations:** 1 Department of Surgery, Inje University College of Medicine, Ilsan Paik Hospital, Goyang 10380, Republic of Korea; 2 Department of Gastrointestinal Surgery, Eunpyeong St. Mary’s Hospital, College of Medicine, The Catholic University of Korea, Seoul 06591, Republic of Korea; 3 Center for Gastric Cancer, National Cancer Center, 323 Ilsan-ro, Ilsanseo-gu, Goyang 10408, Republic of Korea; 4 Department of Surgery, Seoul National University Hospital, Seoul 03080, Republic of Korea; 5 Department of Surgery and Cancer Research Institute, Seoul National University College of Medicine, Seoul National University Hospital, Seoul 03080, Republic of Korea; 6 Cancer Research Institute, Seoul National University College of Medicine, Seoul 03080, Republic of Korea; 7 Department of Surgery, Seoul National University College of Medicine, Seoul National University Bundang Hospital, Seongnam 13620, Republic of Korea; 8 Department of Surgery, Ajou University Hospital, Suwon 16499, Republic of Korea; 9 Department of Surgery, Yonsei University Severance Hospital, Seoul 03722, Republic of Korea; 10 Department of Surgery, Gyeongsang National University Hospital, Daegu 52727, Republic of Korea; 11 Department of Surgery, Kyungpook National University Hospital, Daegu 41566, Republic of Korea; 12 Department of Surgery, Yeouido St. Mary’s Hospital, The Catholic University of Korea, Seoul 14662, Republic of Korea; 13 Department of Surgery, Chonnam National University Medical School, Hwasun 58128, Republic of Korea

**Keywords:** gastrectomy, gastric cancer, pylorus

## Abstract

**Background/Objectives**: During laparoscopic pylorus-preserving gastrectomy (LPPG), the preservation of the infra-pyloric artery (IPA) and dissection of the infra-pyloric lymph node (LN) station 6 are essential, underscoring the importance of understanding the anatomical structure of the IPA. This study aimed to investigate anatomical variations in the IPA and surgical outcomes based on data from a multicenter prospective trial. **Methods**: A post hoc analysis was conducted based on the Korean Laparoendoscopic Gastrointestinal Surgery Study (KLASS)-04 trial, in which patients randomly underwent LPPG or laparoscopic distal gastrectomy (LDG). The IPA variations were categorized into three groups: distal, caudal, and proximal. Clinicopathological characteristics and surgical outcomes were analyzed according to the IPA type. **Results**: Among the 192 patients, the distribution of IPA types was as follows: 45 (23.44%) distal, 74 (38.54%) caudal, and 73 (38.02%) proximal. There were no significant differences in the clinicopathological characteristics between the IPA types. Of the 119 patients who underwent LPPG, a significant difference in operative time was observed based on the IPA type, with a longer duration observed with the distal type compared to that of the proximal type (distal type vs. proximal type: 202.5 (150–275) vs. 170 (105–265) min, *p* = 0.0300). No significant differences were observed in other surgical outcomes. **Conclusions**: The distribution of IPA types was more diverse than that reported in previous studies. There was a statistically significant difference in the operating time based on the IPA type. Identifying IPA variations during LPPG may be beneficial for gastric cancer surgeons.

## 1. Introduction

Gastric cancer ranks as one of the most prevalent malignancies globally, with a particularly high incidence in regions such as East Asia [1,2]. In South Korea, the implementation of a national endoscopy screening program has increased the detection rate of early gastric cancer (EGC), thereby improving the survival rates [3]. Consequently, the interest in EGC treatment has increased, leading to an increasing focus on both performing and researching function-preserving surgeries such as laparoscopic pylorus-preserving gastrectomy (LPPG) [4,5]. Recently, a randomized controlled trial was conducted in Korea evaluating the safety of LPPG in terms of both short-term and long-term surgical and oncological outcomes [6,7].

LPPG is indicated for EGC located in the middle third of the stomach, at least 5 cm from the pylorus [8]. Lymph node (LN) dissection is a critical component of gastric cancer surgery, with the station 6 LN defined as the proximal part of the right gastroepiploic artery (RGEA) and vein [9]. Furthermore, due to the common occurrence of station 6 LN metastasis, station 6 LN resection is mandatory in LPPG [10]. The anastomosis procedure in LPPG requires the preservation of the pylorus, which necessitates ensuring an adequate blood supply. The infra-pyloric artery (IPA) is vital in this regard as it primarily supplies the pyloric antrum [11]. Moreover, IPA variation is closely related to the RGEA, making the dissection of station 6 LNs without damaging the IPA a critical technical aspect of the procedure [12,13].

Although the significance of the IPA is increasingly recognized, existing studies on IPA variation are predominantly based on angiography and autopsy data with limited sample sizes [12,14,15]. The results of these studies also demonstrate varying proportions of IPA variation. Moreover, these studies have primarily focused on investigating the origin of the IPA, without examining how these variations impact surgical outcomes. To date, no studies have comprehensively evaluated the relationship between the IPA and surgical outcomes in LPPG.

This study aimed to identify the types of anatomical variations in the IPA via the post hoc analysis of data from the Korean Laparoendoscopic Gastrointestinal Surgery Study (KLASS), a randomized controlled trial comparing LPPG and laparoscopic distal gastrectomy (LDG). Furthermore, this study aimed to investigate the association between the IPA type and surgical outcomes.

## 2. Materials and Methods

### 2.1. Study Design and Patients

This study was conducted by performing a post hoc analysis of data from the KLASS-04 multicenter randomized clinical trial comparing LPPG and LDG. The patients included in KLASS-04 from July 2015 to July 2017 fulfilled the following criteria: (1) age between 20 and 80 years; (2) Eastern Cooperative Oncology Group (ECOG) performance status score of 0 or 1; (3) pathologically confirmed gastric adenocarcinoma with cT1N0M0 disease stage according to the American Joint Committee on Cancer/Union for International Cancer Control 7th edition; (4) tumors located in the middle third of the stomach, at least 5 cm from the pylorus, and suitable for resection by distal gastrectomy [6,7]. KLASS-04 patients with available data regarding the IPA anatomy were analyzed.

Baseline characteristics, including sex, age, weight, height, body mass index, ECOG performance status, American Society of Anesthesiologists physical status classification, carcinoembryonic antigen levels, and carbohydrate antigen 19-9 levels, were analyzed in patients with available data on the IPA anatomy. Additionally, the surgical outcomes of patients with IPA anatomical data who underwent LPPG were analyzed. These outcomes included the operation time, estimated blood loss, damage to the IPA, damage to the infra-pyloric vein (IPV), distance from the distal margin of the tumor to the pylorus, length of the antral cuff, injury to the hepatic branch of the vagus nerve, preservation of the celiac branch of the vagus nerve, number of dissected stations 6 and 9 LNs, total number of dissected LNs, tumor location, tumor size, proximal margin, distal margin, pathologic T stage, pathologic N stage, and morbidity.

**Figure 1:**
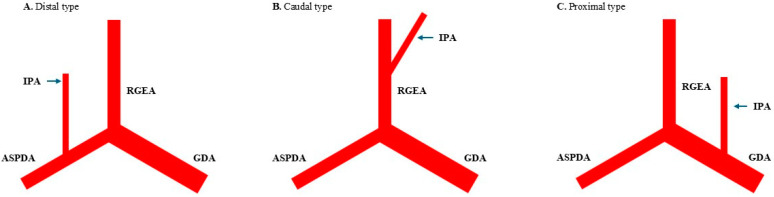
Variation in infra-pyloric artery (IPA). (**A**) Distal type, originating from the anterior superior pancreaticoduodenal artery (ASPDA). (**B**) Caudal type, originating from the right gastroepiploic artery (RGEA). (**C**) Proximal type, originating from the gastroduodenal artery (GDA).

Variations in the IPA were categorized into three types: distal, caudal, and proximal (Figure 1). In this investigation, the distal IPA type originated from the anterior superior pancreaticoduodenal artery (ASPDA), the caudal type originated from the right RGEA, and the proximal type originated from the gastroduodenal artery.

All study procedures adhered to the principles outlined in the 1964 Declaration of Helsinki. The requirement for patient consent was waived due to the retrospective design of the study. This study was approved by the Institutional Review Board of the Seoul National University Hospital (approval number: H-2410-007-1574).

### 2.2. Surgical Quality Control

Only surgeons who had performed at least 50 cases of both LDG and open distal gastrectomy, whose institute had more than 80 cases of gastrectomy per year, and who had performed at least 5 LPPG were eligible to join KLASS-04. At least one unedited video of LPPG was reviewed by 2 or more reviewers with experience in LPPG, using a standardized evaluation sheet.

### 2.3. Statistical Analysis

Continuous variables are expressed as means ± standard deviations or medians with minimum–maximum values, whereas categorical variables are reported as frequencies and percentages. Comparisons of the three groups were conducted using either the chi-squared test for categorical variables, analysis of variance, or the Kruskal–Wallis test for continuous variables, based on Fisher’s exact test and normality test results. In cases where the comparison of the results of the three groups indicated a difference in at least one group, pairwise comparisons were conducted using a post hoc test. All analyses were conducted using SAS version 9.4 (SAS Institute Inc., Cary, NC, USA). *p* < 0.05 was considered statistically significant.

## 3. Results

### 3.1. Patients

**Figure 2:**
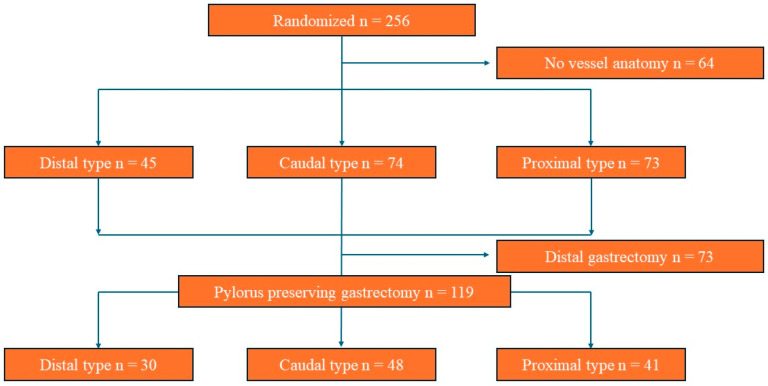
CONSORT diagram.

Among the 256 KLASS-04 patients included in the randomized group from July 2015 to July 2017, 64 were excluded due to missing IPA anatomy data (Figure 2). Of the 192 patients included in the analysis, 45 (23.44%) exhibited the distal type, 74 (38.54%) the caudal type, and 73 (38.02%) the proximal type. No statistically significant differences were observed between the IPA anatomy and the baseline characteristics of the patients (Table 1).

### 3.2. Surgical Outcomes

Among the 119 patients who underwent LPPG, the IPAs in 30 (25.21%) were distal, 48 (40.34%) were caudal, and 41 (34.45%) were proximal (Figure 2). There were no significant differences in the estimated blood loss, damage to the IPA, damage to the IPV, distance from the distal margin of the tumor to the pylorus, length of the antral cuff, preservation of the hepatic branch of the vagus nerve, total number of dissected LNs, tumor location, tumor size, proximal margin, distal margin, pathologic T stage, pathologic N stage, or morbidity based on the IPA type. However, the IPA type significantly impacted the operation time, with the distal type being associated with a longer operative time compared to the proximal type (distal type: 202.5 (150–275) min, proximal type: 170 (105–265) min; *p* = 0.0300). A significant difference was also observed in the number of resected station 9 LNs associated with the IPA type (distal vs. proximal type: 4 (1–9) vs. 2 (0–11); *p* = 0.0112). There was a marginal difference in station 6 LNs among the IPA types (distal type vs. caudal type vs. proximal type: 6 (0–19) vs. 5 (0–16) vs. 7 (0–20); *p* = 0.0879). Furthermore, although not statistically significant, a marginal difference was observed in the number of patients with preserved celiac branches of the vagus nerve according to the IPA anatomy (caudal vs. proximal type: 14.6% vs. 36.3%, *p* = 0.0767) (Table 2).

## 4. Discussion

This study investigated the effect of anatomical variations in the IPA on clinicopathological data and surgical outcomes, specifically in the context of LPPG for EGC. Although several previous studies have focused on variations in the IPA [14,15], this is the first study to analyze IPA variations with regard to surgical outcomes using data from a randomized prospective multicenter study (KLASS-04).

In our study, the least common distribution of IPA vessels was the distal type (23.44%), with the caudal (38.54%) and proximal (38.02%) types exhibiting similar proportions. In the LPPG group, the distal type was the least common at 25.21%, while the caudal and proximal types accounted for 40.34% and 34.45%, respectively. Our results differ from the findings of previously published studies. In one meta-analysis, contrary to our study, the distal type (40.4%) was the most common, while the proximal type (23.7%) accounted for the smallest proportion [12]. Two other studies conducted in Japan [14] and China [15] also reported different proportions of IPA vessel types. In Japan, the order was distal (64.2%), caudal (23.1%), and proximal (12.7%), whereas, in China, it was proximal (35.8%), distal (31.0%), and caudal (27.2%). The reasons for the differing proportions of IPA variations are not clearly understood. However, one study reported a significant association with geographic factors, suggesting that genetic factors may exert an influence [15].

In our study, a significant difference was observed in the operative time according to the IPA variation, which may be attributed to the surgical technique employed during LPPG. When performing LPPG, it is crucial to avoid injury to the IPA, which supplies blood to the prepyloric antrum for the functional preservation of the pylorus [16]. Therefore, during the dissection of the station 6 LN, the IPA was identified by following the route from the gastroduodenal artery along the RGEA to the ASPDA [17,18]. The more proximally located IPA facilitates more rapid identification, enabling the earlier ligation of the RGEA and dissection of the station 6 LN. Consequently, the operative time was shorter for the proximal type and longer for the distal type. Consistent with this observation, our study revealed that the proximal type had the shortest operative time, followed by the caudal type, with the distal type exhibiting the longest operative time. The post hoc difference in the operative time between the proximal and distal types was statistically significant (distal vs. proximal type: 202.5 (150–275) vs. 170 (105–265) min; *p* = 0.0300). Considering that the operative time is one of the components of the Estimation of Physiologic Ability and Surgical Stress (E-PASS) score, which is used to predict postoperative complications and mortality, the variation in the operative time according to the IPA anatomy may potentially influence postoperative outcomes, including complications or mortality [19]. Although no significant difference in morbidity was observed according to the IPA anatomy in this study, further large-scale studies may be necessary to validate these findings.

In EGC, station 6 LN dissection is considered crucial when performing pylorus-preserving gastrectomy because of the risk of LN 6 metastasis [10,20]. The station 6 LNs can be further subdivided into 6v (proximal part of the RGEV), 6a (RGEA), and 6i (infra-pyloric vessel) nodes [21]. Dissecting the 6i LN is the most technically challenging aspect because it must be performed without injuring the IPA. In the proximal type, the IPA is identified first, enabling the ligation of the RGEA, which facilitates the faster and easier dissection of the 6a and 6v LNs. Conversely, in the distal type, the IPA is identified last and requires careful dissection to avoid vessel injury, thereby rendering the procedure more technically challenging. In our study, the difference was marginally significant; it may be one of the reasons that more LNs were harvested in the proximal type (distal type vs. caudal type vs. proximal type: 6 (0–19) vs. 5 (0–16) vs. 7 (0–20); *p* = 0.0879). From an alternative perspective, the absence of a statistically significant difference in the number of resected LNs based on the vessel anatomy in the KLASS-04 study could serve as evidence of a well-controlled study.

The number of resected station 9 LNs was lower in the distal type than in the proximal type (distal vs. proximal type: 4 (1–9) vs. 2 (0–11); *p* = 0.0112). Furthermore, there was a statistically marginal tendency to preserve the vagus nerve celiac branch more in the proximal type than in the caudal type (caudal vs. proximal type: 14.6% vs. 36.3%; *p* = 0.0767). Although the exact reason for the association between the IPA type and the number of resected station 9 LNs is unclear, the statistically significant findings related to LN 9 and the preservation of the vagus nerve celiac branch suggest that other specific surgical factors may also be involved. Further analysis of the surgical outcomes is necessary.

No statistically significant association was observed between the presence of IPA and IPV injury and the type of IPA vessel in this study (*p* > 0.9999). However, because the data used in this study were derived from patients who underwent LPPG, there were very few cases of injury, with one patient experiencing an IPA injury and four patients experiencing an IPV injury. Although no studies have been conducted on this topic to date, our findings suggest that, as the surgical time increases with the distal type, IPA or IPV injuries may be more likely.

This study has several limitations. First, although there were 256 patients, the absence of data for 64 significantly reduced the number of patients analyzed. However, among the 64 cases with missing data, the majority (55 cases) belonged to the LDG group. Therefore, the impact on the analysis of the short-term surgical outcomes was minimal. Second, as this was a well-controlled study, few patients experienced vessel injury. Third, this study did not include analyses of long-term surgical outcomes, oncological safety, or quality of life. Lastly, this study was conducted in a single geographic region in Korea. Nevertheless, the observed differences in the operative time and number of dissected LNs according to the IPA type in our study suggest that prior knowledge of the IPA type in advance could be beneficial for surgical planning. Further multicenter, large-scale studies involving diverse geographic regions will be necessary in the future.

## 5. Conclusions

In conclusion, the present study exhibited greater diversity in the distribution of IPA types compared to previous studies. A statistically significant difference was observed in the operating time and dissected station 9 LNs depending on the IPA type. Identifying IPA variations during LPPG may therefore be beneficial for gastric cancer surgeons, with the potential to enhance the precision and safety of surgical procedures and positively impact patient outcomes. Further research is warranted to clarify the differences in operation times depending on the IPA type.

## Figures and Tables

**Table 1:** Baseline characteristics of patients.

Variable		ASPDA (Distal) N = 45	RGEA (Caudal) N = 74	GDA (Proximal) N = 73	*p*-Value
**Sex**	Female	24 (53.3%)	36 (48.7%)	42 (57.5%)	0.5582 †
	Male	21 (46.7%)	38 (51.4%)	31 (42.5%)	
**Age**	Mean ± sd	55.5 ± 11.0	58.4 ± 10.8	55.3 ± 10.1	0.1621 *
**Weight**	Mean ± sd	61.6 ± 9.9	61.0 ± 11.4	62.8 ± 9.7	0.5600 *
**Height**	Mean ± sd	161.7 ± 8.3	160.9 ± 8.9	162.6 ± 7.8	0.5005 *
	Median (min–max)	161.3 (140–178.7)	161.2 (137.6–180.7)	162.7 (146.6–179)	
**BMI**	Mean ± sd	23.5 ± 3.0	23.4 ± 2.9	23.7 ± 2.5	0.8149 *
**ECOG**	0	45 (100%)	71 (96%)	72 (98.6%)	0.4520 ‡
	1	0 (0%)	3 (4.1%)	1 (1.4%)	
**ASA**	1	24 (53.3%)	47 (63.5%)	48 (65.8%)	0.3900 ‡
	2	21 (46.7%)	27 (36.5%)	24 (32.9%)	
	3	0 (0%)	0 (0%)	1 (1.4%)	
**CEA**	Median (min–max)	1.7 (0.3–12.9)	1.6 (0.5–8)	1.3 (0.5–8)	0.1284 #
**CA 19-9**	Median (min–max)	6.7 (0.2–39.9)	6.8 (0.6–119.8)	6 (0.9–40.6)	0.8074 #

†: Chi-squared test, ‡: Fisher’s exact test, *: analysis of variance (ANOVA), #: Kruskal–Wallis test. ASPDA, anterior superior pancreaticoduodenal artery; RGEA, right gastroepiploic artery; GDA, gastroduodenal artery; ECOG, Eastern Cooperative Oncology Group; ASA, American Society of Anesthesiologists; CEA, carcinoembryonic antigen; CA 19-9, carbohydrate antigen 19-9.

**Table 2:** Surgical outcomes.

Variable		ASPDA (Distal)	RGEA (Caudal)	GDA (Proximal)	*p*-Value	Post Hoc		
		N = 30	N = 48	N = 41		ASPDA vs. RGEA	ASPDA vs. GDA	RGEA vs. GDA
Operating time (min)	Median (min–max)	202.5 (150–275)	195 (110–275)	170 (105–265)	0.0265 **#**	0.7240	0.0300	0.2234
Estimated blood loss (cm^3^)	Median (min–max)	39.3 (10–1000)	38.4 (5–220)	35.5 (5–785.6)	0.7512 #	>0.9999	>0.9999	>0.9999
IPA injury	No	30 (100%)	47 (97.9%)	41 (100%)	>0.9999 ‡	>0.9999	>0.9999	>0.9999
	Yes	0 (0%)	1 (2.1%)	0 (0%)				
IPV injury	No	29 (96.7%)	46 (95.8%)	40 (97.6%)	>0.9999 ‡	>0.9999	>0.9999	>0.9999
	Yes	1 (3.3%)	2 (4.2%)	1 (2.4%)				
Length from tumor to pylorus (cm)	Median (min–max)	7.8 (4.5–20)	7 (4–15)	8 (3–18.5)	0.9398 #	>0.9999	>0.9999	>0.9999
Antral cuff length (cm)	Median (min–max)	4 (3–8.6)	4 (3–6)	4 (3–6)	0.3724 #	0.5285	>0.9999	>0.9999
Vagus nerve hepatic branch injury	No	29 (96.7%)	48 (100%)	39 (95.1%)	>0.9999 ‡	>0.9999	>0.9999	>0.9999
	Yes	1 (3.3%)	0 (0%)	2 (4.9%)				
Vagus nerve celiac branch preserved	No	23 (76.7%)	41 (85.4%)	26 (63.4%)	0.0542 †	>0.9999	0.9060	0.0767
	Yes	7 (23.3%)	7 (14.6%)	15 (36.6%)				
Tumor location	Upper	0 (0%)	1 (2.1%)	0 (0%)	0.0923 ‡	0.1492	>0.9999	0.2337
	Middle	21 (70%)	42 (87.5%)	30 (75%)				
	Low	9 (30%)	5 (10.4%)	10 (25%)				
Tumor size (mm)	Median (min–max)	19 (2.3–57)	20 (0.8–75)	18 (0–64)	0.9825 #	>0.9999	>0.9999	>0.9999
Proximal margin (cm)	Median (min–max)	2.8 (0.2–7.3)	1.95 (0.5–6.5)	2.2 (0.3–13.5)	0.1074 #	0.1239	>0.9999	0.4993
Distal margin (cm)	Median (min–max)	2.75 (0.8–10)	3.5 (0.2–11)	2.3 (0.2–13.8)	0.3906 #	>0.9999	>0.9999	0.5234
Resected LN station 6	Median (min–max)	6 (0–19)	5 (0–16)	7 (0–20)	0.0879 #	0.3954	>0.9999	0.1030
Resected LN station 9	Median (min–max)	4 (1–9)	3 (0–10)	2 (0–11)	0.0110 #	0.2687	0.0112	0.1598
Resected total LN	Median (min–max)	34.5 (18–88)	34 (15–82)	39 (16–65)	0.1164 #	0.5965	>0.9999	0.1256
pTstage	T1a	20 (66.7%)	28 (58.3%)	25 (62.5%)	0.9780 ‡	>0.9999	>0.9999	>0.9999
	T1b	9 (30%)	18 (37.5%)	14 (35%)				
	T2	1 (3.3%)	1 (2.1%)	1 (2.5%)				
	T3	0 (0%)	1 (2.1%)	0 (0%)				
pNstage	N0	26 (86.7%)	43 (89.6%)	37 (90.2%)	0.8886 ‡	>0.9999	>0.9999	>0.9999
	N1	3 (10%)	4 (8.3%)	3 (7.3%)				
	N2	1 (3.3%)	0 (0%)	1 (2.4%)				
	N3b	0 (0%)	1 (2.1%)	0 (0%)				
Morbidity	No	22 (73.3%)	39 (83%)	35 (85.4%)	0.4088 †	>0.9999	0.7174	>0.9999
	Yes	8 (26.7%)	8 (17%)	6 (14.6%)				

†: Chi-squared test, ‡: Fisher’s exact test, #: Kruskal–Wallis test. ASPDA, anterior superior pancreaticoduodenal artery; RGEA, right gastroepiploic artery; GDA, gastroduodenal artery; IPA, infra-pyloric artery; IPV, infra-pyloric vein; LN, lymph node.

## Data Availability

Data are available from the authors upon request.
